# Comprehensive integrated NGS-based surveillance and contact-network modeling unravels transmission dynamics of vancomycin-resistant enterococci in a high-risk population within a tertiary care hospital

**DOI:** 10.1371/journal.pone.0235160

**Published:** 2020-06-24

**Authors:** Bernd Neumann, Jennifer K. Bender, Benjamin F. Maier, Alice Wittig, Stephan Fuchs, Dirk Brockmann, Torsten Semmler, Hermann Einsele, Sabrina Kraus, Lothar H. Wieler, Ulrich Vogel, Guido Werner

**Affiliations:** 1 Division of Nosocomial Pathogens and Antibiotic Resistance, Robert Koch Institute, Wernigerode, Germany; 2 Computational Epidemiology, Robert Koch Institute, Berlin, Germany; 3 Department of Physics, Humboldt University of Berlin, Berlin, Germany; 4 Institute for Theoretical Biology, Humboldt University of Berlin, Berlin, Germany; 5 Microbial Genomics, Robert Koch Institute, Berlin, Germany; 6 Department of Internal Medicine II, University Hospital Würzburg, Wüzburg, Germany; 7 Robert Koch Institute, Berlin, Germany; 8 Institute for Hygiene and Microbiology, Julius-Maximilians University Würzburg, Würzburg, Germany; Hospital Universitario Ramon y Cajal, SPAIN

## Abstract

Vancomycin-resistant *E*. *faecium* (VRE) are an important cause of nosocomial infections, which are rapidly transmitted in hospitals. To identify possible transmission routes, we applied combined genomics and contact-network modeling to retrospectively evaluate routine VRE screening data generated by the infection control program of a hemato-oncology unit. Over 1 year, a total of 111 VRE isolates from 111 patients were collected by anal swabs in a tertiary care hospital in Southern Germany. All isolated VRE were whole-genome sequenced, followed by different in-depth bioinformatics analyses including genotyping and determination of phylogenetic relations, aiming to evaluate a standardized workflow. Patient movement data were used to overlay sequencing data to infer transmission events and strain dynamics over time. A predominant clone harboring *vanB* and exhibiting genotype ST117/CT469 (n = 67) was identified. Our comprehensive combined analyses suggested intra-hospital spread, especially of clone ST117/CT469, despite of extensive screening, single room placement, and contact isolation. A new interactive tool to visualize these complex data was designed. Furthermore, a patient-contact network-modeling approach was developed, which indicates both the periodic import of the clone into the hospital and its spread within the hospital due to patient movements. The analyzed spread of VRE was most likely due to placement of patients in the same room prior to positivity of screening. We successfully demonstrated the added value for this combined strategy to extract well-founded knowledge from interdisciplinary data sources. The combination of patient-contact modeling and high-resolution typing unraveled the transmission dynamics within the hospital department and, additionally, a constant VRE influx over time.

## Introduction

Enterococci are ubiquitous environmental gram-positive bacteria naturally colonizing the gastrointestinal tract of animals and humans [1]. Some species, in particular *Enterococcus faecalis* and *Enterococcus faecium*, act as opportunistic pathogens and can cause severe infections leading to bacteremia or endocarditis [2–4]. The treatment of enterococcal infections is frequently limited due to a multitude of intrinsic and acquired antibiotic resistances [1, 2, 5, 6]. The ability of enterococci to withstand harsh environmental conditions, such as desiccation, and to form biofilms on abiotic surfaces increases the necessity of environmental decontamination [7, 8]. For instance, hospital patients could acquire vancomycin-resistant *E*. *faecium* (VRE) through exposure to VRE-contaminated surfaces and hospital facilities [9, 10]. In particular, resistance to the important therapeutic agent vancomycin represents a challenge for a successful treatment of *Enterococcus* spp. [6, 11]. Currently, nine different vancomycin resistance operons (*van*), which mediate vancomycin resistance on varying levels, are known; the most prevalent types in *E*. *faecium* are *vanA* and *vanB*, both of which are transferable through mobile genetic elements [5, 11, 12].

Since 2014, a shift from *vanA* to *vanB* type VRE has been observed in German clinical settings [11, 13]. Both genotypes can be presented by *E*. *faecium* lineages; however, the German National Reference Centre for Staphylococci and Enterococci has noted highly prevalent sequence types (ST) that are predominantly associated with either *vanA* or *vanB*: ST117 (*vanB*), ST203 (*vanA*), and ST80 (*vanB*) [14]. In contrast to *E*. *faecalis*, distinct lineages for animals, human colonization, and hospital-association exist within the species *E*. *faecium* [13, 15, 16]. *Enterococcus faecium* strains of clade B have been described as human colonizers of the gastro intestinal tract, whereas *E*. *faecium* strains of clade A represent hospital-adapted and infection-associated lineages [17].

Noteworthy, infection surveillance revealed a strong increase of vancomycin resistance in enterococcal infections observed in intensive care units and surgical departments in Germany over the past 10 years [18, 19]. This trend is worrisome, especially for hematology and oncology units, as outbreaks of infections within a vulnerable patient population are frequently caused by enterococci [20]. Patients with hematological malignancies are at high risk for acquiring healthcare-associated infections by VRE. In these patients, impairments of the gut microbiome by frequent antibiotic treatment may result in an “overgrowth” of VRE, hence leading to an increased risk for enterococcal (VRE) bloodstream infections [21–24]. Contaminated patient rooms, healthcare workers, as well as contact between patients are of particular importance for transmitting VRE within a hospital [10, 25–28]. More recent studies highlighted the advantages of using data-driven approaches to measure the effects of contact monitoring in order to reveal pathogen spread in hospitals, further using these data to assess infection control and prevention strategies [29, 30].

Another major challenge in controlling VRE outbreaks is the differentiation between patient colonization, infection, and the role of environmental contamination. Therefore, combining high-resolution typing with reliable clinical and epidemiological data is indispensable for a valid assessment of putative outbreak situations [31]. Typing by macro-restriction analysis and subsequent pulsed-field gel electrophoresis (PFGE) has been the standard method, but lacks international standardization; recent analyses revealed discrepancies to investigations based on whole-genome sequencing (WGS) [32]. In contrast to PFGE, multi-locus sequence typing (MLST) provides an international and expandable typing nomenclature, but its resolution is limited due to the highly clonal nature of VRE in healthcare settings [13, 15].

Next-generation sequencing (NGS) enables high-throughput analyses of entire bacterial genomes at affordable costs and has become a useful approach for outbreak and population analyses [33–37]. One common pathway for NGS-derived data analyses is based on reference-dependent alignment of raw sequence data, followed by variant calling to deduce single nucleotide polymorphisms (SNPs) for downstream analyses [11, 38–40]. However, the SNP-based genotyping relies on an appropriate reference genome and, as PFGE, lacks a standardized nomenclature. This is bypassed by applying a so-called core genome MLST (cgMLST), which combines the extensive data generated by WGS with a gene target-based typing such as MLST, but on a scale of hundreds of gene targets. Thereby, a robust and expendable database and international nomenclature for genotyping are provided [33, 41]. Although bioinformatics tools are a prerequisite to handle and interpret NGS-derived data, appropriate approaches of data analysis are still not standardized and under constant evaluation and discussion [40, 42].

In this study, we applied NGS and overlaid the obtained results with data of patients’ movement in order to broaden the insights into VRE population and transmission dynamics in a tertiary care hospital and within a high-risk patient population. In particular, we applied and assessed different bioinformatics approaches for NGS-based data analyses and phylogenetic tree building. Furthermore, we constructed a data-driven, dynamic contact network model to identify and exclude potential transmissions between patients and transmission cascades. Altogether, by combining these different approaches in an interdisciplinary fashion, we were able to provide (i) transmission hotspots with a better resolution and thus (ii) a suitable strategy to prevent further dissemination of VRE.

## Materials and methods

### VRE sampling

Patients admitted to the clinic specialized in hematology, oncology, stem cell therapy, gastroenterology, hepatology, infectious diseases, and rheumatology in a tertiary care hospital in Southern Germany were screened for VRE by perianal swabbing on admission, weekly and upon discharge. The screening is part of a continuously applied routine infection control program of this unit. The sampled bacteria were enriched in BBL Enterococcosel broth (Becton Dickinson, Franklin Lakes, NJ, USA) supplemented with vancomycin (6 mg/L). In case of suspicious growth, the bouillon was plated on Brilliance™ VRE agar (Oxoid/Thermo Fisher Scientific, Basingstoke, UK). The VRE obtained in the routine program between July 1, 2014 and June 30, 2015 were analyzed by NGS.

### Study design

Routine screening of high-risk patients treated in the clinic for hematology and oncology was conducted as part of the infection control program of the university hospital, which was put in place by the infection control panel. This routine screening program is continued to date. The retrospective observational study of VRE-strains and movement of VRE-positive patients described herein was conducted to assess the efficacy of infection control policies and to deduce measures for quality improvement as part of the hospital’s quality improvement efforts. The infection control policy with regards to VRE was comprised during the observation period routine screening for VRE carriage, single room placement, and contact isolation of VRE-positive patients and enhanced environmental cleaning and decontamination. Hand hygiene was regularly reinforced and trained by infection control nurses; however, compliance was not quantified by standardized observation. Patient movement data were extracted from the hospital information system by the hospital administration. Patient and case-identification numbers were used throughout the study. As the observational study used routine data from the infection control program and data were processed anonymously, the need to involve the ethics committee was neglected.

### Determination of antimicrobial susceptibility

The obtained VRE strains were tested for susceptibility to 18 antibiotics, using the broth microdilution method according to EUCAST standards and breakpoints (v 8.0; as of December 1, 2018, www.eucast.org) or epidemiological cut-off values when no breakpoints were defined, as described before [43].

### Genomic library preparation and next-generation sequencing

The bacterial DNA was extracted using the DNeasy Blood and Tissue kit according to the protocol of the manufacturer (Qiagen, Hilden, Germany). Quantification was carried out using the Qubit dsDNA HS Assay Kit (Invitrogen/Thermo Fisher Scientific, Karlsruhe, Germany). Library preparation with 1 ng of extracted DNA was done by applying the Nextera XT DNA Library Prep Kit according to the manufacturer’s instructions (Illumina, San Diego, CA, USA). Libraries were subjected to whole-genome sequencing using an Illumina^®^ MiSeq benchtop devise (Illumina, San Diego, CA, USA) in paired-end mode (v3 chemistry; 2 x 300 bp) as described elsewhere [11].

### Reference-based read alignment and SNP calling

The raw reads were trimmed using Trimmomatic (v. 0.32; default parameters with sliding window 4:15), and the resulting paired-end reads were aligned to the completed reference sequence *E*. *faecium* 64/3 (NZ_CP012522.1/CP012522.1) using BWA-SW (v. 0.7.13-r1126; default parameters) [44, 45]. Subsequent variant calling was performed using VarScan (v. 2.3; default parameters) [46]. Due to the high genetic recombination rates in the *E*. *faecium* genome, SNPs with a distance of 300 or less to each other were excluded by applying SNPfilter (v. 2.2.0; exclusion distance d = 300) [39]. Maximum likelihood phylogenetic trees were generated on the basis of retained SNPs using RAxML with a GTR GAMMA nucleotide model (v. 8.2.7; bootstrap: 1,000 permutations) [47].

### *De novo* sequence reconstruction

For *de novo* sequence reconstruction, raw reads were trimmed using Trimmomatic (v. 0.32; default parameters with maxinfo 50:0.8) [44]. The A5-miseq algorithm (v. 20150522, default parameters) was applied to assemble trimmed paired read data [48].

### Sequence data-based genotyping

The *de novo* reconstructed sequences were used to extract sequence types (ST) by using the SeqSphere^+^ software (v. 4.1.9 Ridom GmbH, Münster, Germany), which accesses the public MLST scheme for *E*. *faecium*, which is available on the PubMLST website (www.pubmlst.org/efaecium/) [49, 50]. For further high-resolution genotyping, the cgMLST *E*. *faecium* scheme of the SeqSphere^+^ software was applied [33]. Discrete complex types (CT) were deduced for each sequenced isolate. On basis of the cgMLST analyses, a neighbor-joining phylogenetic tree and, further, a minimum spanning tree were calculated in SeqSphere^+^.

### Maximum common genome analyses

As another alternative approach to investigate the phylogenetic relatedness within the strain collection, the maximum common genome (MCG) approach was performed [51]. The MCG is based on an orthologous gene set which is present in all the genomes of the entire strain collection investigated. First, clustering for similarity was performed on the predicted coding sequences with a minimum sequence similarity on the nucleotide level of 70% and coverage of at least 90% for candidate genes to be included in the MCG (n = 1,811). In a next step, the allelic variants were extracted from the genomes by using a BLAST-like approach with an in-house pipeline. All variants for each gene in the MCG were aligned individually with MUSCLE and concatenated to the final MCG alignment [52]. Based on this core gene alignment, a phylogenetic tree was calculated using RAxML with a GTR GAMMA nucleotide model (v. 8.2.7; bootstrap: 100 permutations) [47].

### Alignment-free approach for phylogenetic analyses

All reconstructed sequences were also subjected to an alignment-free approach using feature frequency profiling (FFP) [53]. This approach represents a mere bioinformatics way to analyze the relatedness of the isolates. The FFP algorithm counts frequencies of *l*-mers of a defined length within the genome sequences. First, the length of *l*-mers was determined by calculating the lower and upper limits using one example sequence. After this, the FFP algorithm was run on all reconstructed sequences from the *de novo* assembly. Within the process the sequences were dissected to *l*-mers, followed by profiling and normalization steps, resulting in the calculation of a distance matrix and a neighbor-joining phylogenetic tree.

### Phylogenetic tree visualization

All calculated phylogenetic trees were visualized using the free online tool iTOL (v. 5.5; http://itol.embl.de) [54].

### Comparison of phylogenetic trees

The phylogenetic trees from the different approaches were used to compare the concordance of the methods by two major parameters. The first parameter represents the tree topology and isolates clustering, while the second parameter examines the branch length which correlates with the phylogenetic distances between isolates. Using the normalized absolute difference of patristic distances of isolate pairs from each of the phylogenetic analyses, an isolate-by-isolate comparison was performed for all applied tree-building methods [41]. To generate distance values suitable to visual compare the different approaches, it was necessary to multiply the absolute differences by 1 x 10^9^ and to transform the obtained values (logarithm with basis = 2). The resulting values were used as a measure of grade of concordance and visualized by heatmaps, using iTOL (v. 5.5) [54].

### Patient movement data visualization

Patient data included spatial and temporal information about admission, discharge, and movement within the hospital as well as the time of their first positive VRE isolation. For each patient, ward- and room-numbers were available. Data were extracted from the hospital information system as part of an evaluation of the efficacy of the infection control policy applied. No further personal data were extracted. To monitor and infer putative transmission events quickly, patient movements within the hospital were compared to genotyping results. To this end, an interactive visualization tool was developed, which illustrates a timeline of movements for each patient. For the corresponding VRE isolates, genotypes were depicted, and an SNP-based phylogenetic tree was connected to the timeline. Patients that stayed in the same rooms were visually connected.

### Patient contact network analysis

To improve the transmission event inference based on molecular genotyping analyses, a patient-contact network model was constructed from patient movement within the hospital. The model accounts for room colonization with VRE by colonized patients. Implicitly, the model captures spreading between rooms. Colonized rooms can spread VRE to susceptible patients. Along this line of reasoning, we aimed to estimate the total amount of time in which a source patient may be responsible for the colonization of a target patient, called the “infectious-contact time”. The “infectious-contact time” increases when the hospital stays of two patients are close in time and in physical space (i.e., in the same room or the same ward). Two free parameters control (a) the weight of spatial proximity, e.g., the proximity of hospital rooms, and (b) the influence of temporal proximity as the time it takes a colonized room to be considered VRE-free after a colonized patient left. A detailed derivation of the infectious-contact time is provided in S1 Appendix.

Close physical proximity is one of the main risks of acquiring VRE colonization. For modelling, we assumed that sharing a room with a colonized patient yields a 10 times higher infectious-contact time than staying in the same ward but in different rooms. Analogously, staying in different rooms of the same ward yields a 10 times higher infectious-contact time than staying in different wards.

Patients were at higher risk if their rooms had colonized pre-occupants. A room’s colonization status was modeled as linearly decreasing in time; beginning at the time a patient leaves the room. The linear decay lasts until a recovery period has passed, after which the room is assumed to be entirely recovered. We set this recovery time to 3 d, based on a study showing that on average 2.8 room cleanings are necessary to eradicate VRE from a hospital room and on the fact that the hospital rooms were completely cleaned on a daily basis. Respecting the high general enterococcal tolerance to survive in the hospital environment, a maximum recovery time of 28 d and an adjusted time of 10 d due to routine cleaning were used for modeling of transmission networks. As VRE screenings were conducted on a weekly basis and an incubation period of 2 d was assumed, a target patient was set to be susceptible for colonization only within 7 d prior to the 2 d before their positive VRE test. This period will be referred to as “patient incubation time”. Patients that were tested as VRE-positive 2 d after admission were taken into consideration as positive at admission and therefore as influx into the hospital [55].

## Results

### Patient cohort

The strain collection resulted from a routine VRE-screening program of a German tertiary care hospital treating patients with hematologic and oncologic malignancies, including bone marrow and stem cell transplant patients. During the period (July 1, 2014 to June 30, 2015), 2,015 patients were admitted to the hospital wards and a total of 1,606 patients were screened (79.7%). Of those, 111 patients (6.9%) were positive for VRE. There was no VRE bacteremia or urinary tract infection. During the observation period, VRE were detected in clinically relevant samples from only two patients; a solitary detection was in a peripheral venous catheter, but with less than 15 colony-forming units and without a positive blood culture. The second patient was admitted with VRE, and consecutively, VRE was isolated from ascites. In total, 2,004 patient days of VRE patients were audited, which were counted irrespective of the time of positivity. The 111 VRE strains of the positively tested patients were kept, and thus represented the isolate collection for the present study.

### Typing, phylogeny, and comparative bioinformatics analyses

**Fig 1:**
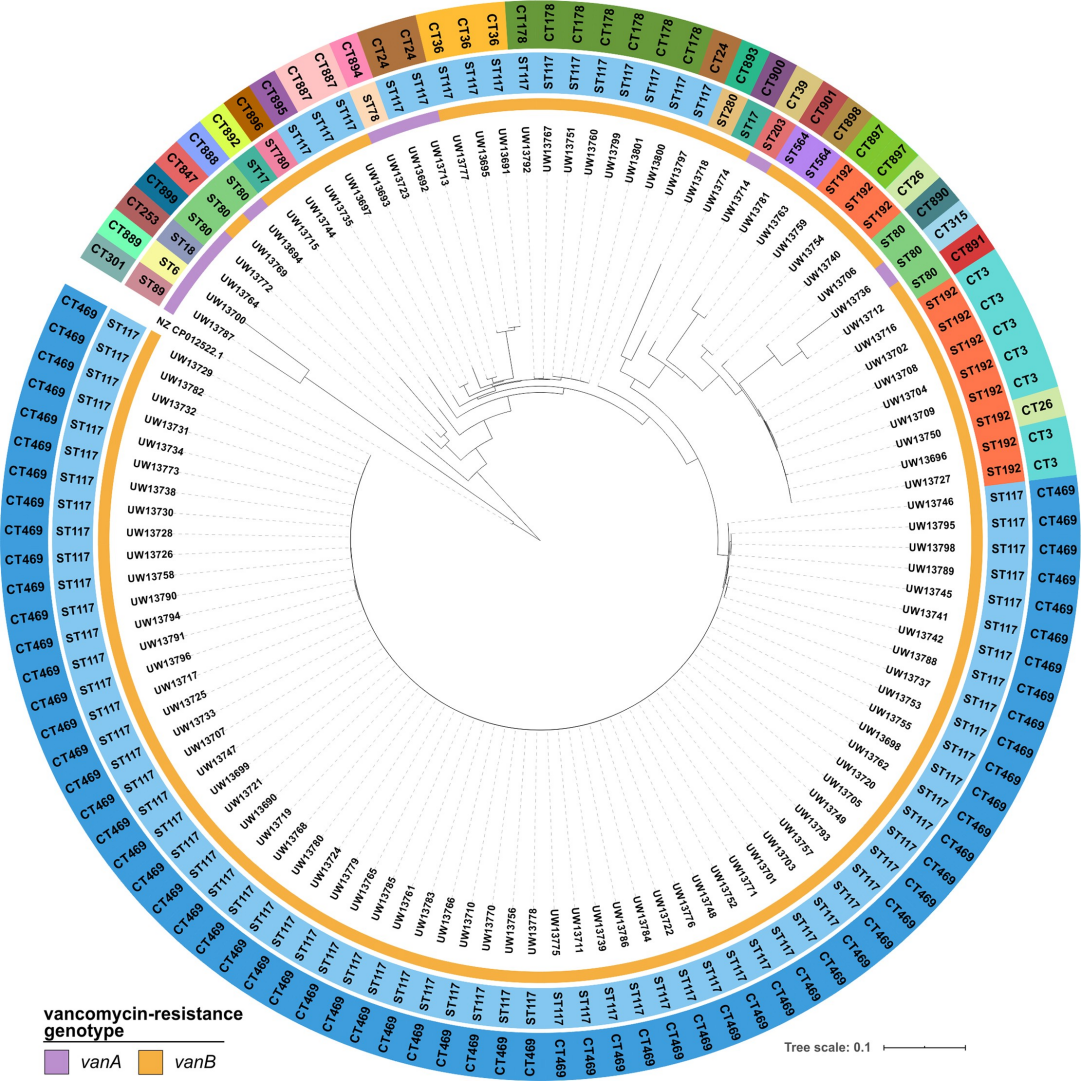
Studied collection of *E*. *faecium* from the patient screening in a hematology and oncology department (n = 111). The phylogenetic tree is the result of applying the SNP approach and based on read-alignment and variant calling. Available data for sequence types (ST; middle circle) and complex types (CT; outer circle) are colored accordingly. The distribution of the vancomycin-resistance genotypes *vanA* and *vanB* is depicted (see legend). The strain *E*. *faecium* 64/3 (NZ_CP012522.1) was used as a reference for read alignment (no color). Visualization was realized using iTOL.

**Fig 2:**
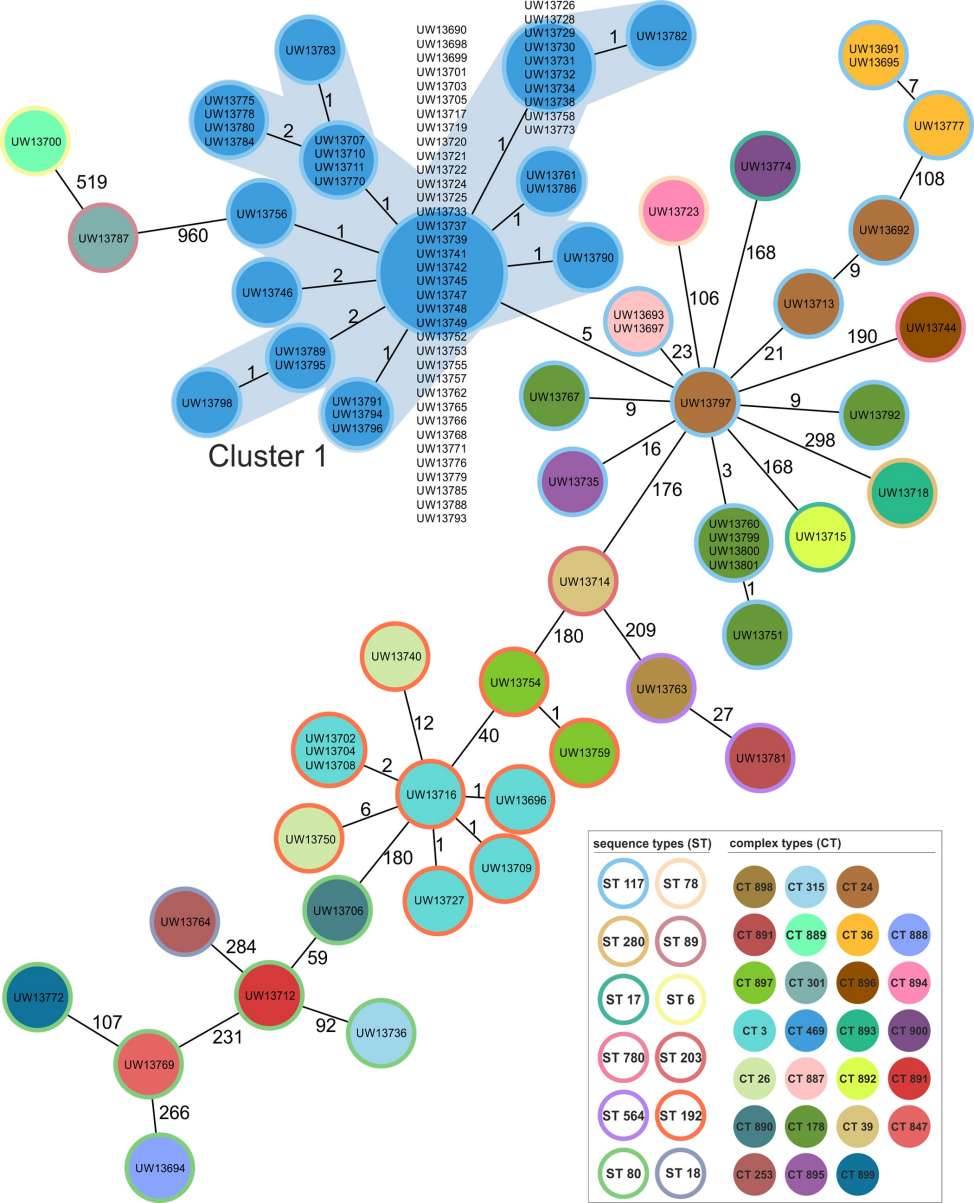
Minimum spanning tree of the entire isolate collection of *E*. *faecium* from the patient screening in a hematology and oncology department (n = 111). The tree is based on cgMLST analyses and was calculated using the SeqSphere^+^ software. Each circle represents one allele profile. If more than one strain exhibits an identical profile, the size of the circle is adapted. The number on the connecting lines represents the allele differences between two isolates. Color-codes represent distinct sequence types and also the corresponding complex types (see legend). Cluster 1 is visualized by grey shading and depicts the predominant clone with the ST117/CT469 genotype.

All isolates of this study were confirmed as VRE, whereby 91% belonged to the *vanB* and 9% to the *vanA* genotype (Fig 1). None of the isolates showed resistance to the last-line antibiotics daptomycin, linezolid, or tigecycline. Further, the entire isolate collection was subjected to WGS, and the obtained sequence data were used for different typing approaches and phylogeny reconstruction (Fig 1). Sequence types (ST) and cgMLST-based complex types (CT) were extracted from genome sequences reconstructed by *de novo* assembly. The study isolate collection comprised 12 different STs (Table 1), whereby ST117 (n = 83) showed the highest frequency followed by ST192 (n = 11) and ST80 (n = 6). By applying the cgMLST scheme, 26 CTs were observed (Table 1). Novel CTs identified (n = 15) as part of this study were submitted to the cgMLST database (www.cgMLST.org), using the SeqSphere^+^ software. The majority of the ST117 isolates belonged to CT469 (n = 67), which grouped together in a distinct cluster in the cgMLST-based minimum spanning tree (Fig 2). The abundance of isolates exhibiting ST117/CT469 was 60.4% for the entire study collection, and all exhibited the *vanB* genotype (Fig 1, Table 1).

**Table 1:** List of *E*. *faecium* isolates obtained by a one year routine screening patients admitted to a hematological/oncological department in a German hospital.

Strain ID	Year of Isolation	Sequence Type (ST)	Complex Type (CT)	*van*- Genotype	Patient ID^1^
UW13690	2014	117	469	*vanB*	0
UW13691	2014	117	36	*vanB*	1
UW13692	2014	117	24	*vanA*	2
UW13693	2014	117	887	*vanB*	3
UW13694	2014	80	888	*vanA*	4
UW13695	2014	117	36	*vanB*	n.d.
UW13696	2014	192	3	*vanB*	5
UW13697	2014	117	887	*vanB*	6
UW13698	2014	117	469	*vanB*	7
UW13699	2014	117	469	*vanB*	8
UW13700	2014	6	889	*vanA*	n.d.
UW13701	2014	117	469	*vanB*	9
UW13702	2014	192	3	*vanB*	10
UW13703	2014	117	469	*vanB*	11
UW13704	2014	192	3	*vanB*	12
UW13705	2014	117	469	*vanB*	13
UW13706	2014	80	890	*vanB*	14
UW13707	2014	117	469	*vanB*	15
UW13708	2014	192	3	*vanB*	16
UW13709	2014	192	3	*vanB*	17
UW13710	2014	117	469	*vanB*	18
UW13711	2014	117	469	*vanB*	19
UW13712	2014	80	891	*vanB*	n.d.
UW13713	2014	117	24	*vanA*	20
UW13714	2014	203	39	*vanA*	21
UW13715	2014	17	892	*vanB*	22
UW13716	2014	192	3	*vanB*	23
UW13717	2014	117	469	*vanB*	n.d.
UW13718	2014	280	893	*vanB*	24
UW13719	2014	117	469	*vanB*	25
UW13720	2014	117	469	*vanB*	26
UW13721	2014	117	469	*vanB*	27
UW13722	2014	117	469	*vanB*	28
UW13723	2014	78	894	*vanA*	29
UW13724	2014	117	469	*vanB*	30
UW13725	2014	117	469	*vanB*	31
UW13726	2014	117	469	*vanB*	32
UW13727	2014	192	3	*vanB*	33
UW13728	2014	117	469	*vanB*	34
UW13729	2014	117	469	*vanB*	35
UW13730	2014	117	469	*vanB*	36
UW13731	2014	117	469	*vanB*	37
UW13732	2014	117	469	*vanB*	38
UW13733	2014	117	469	*vanB*	39
UW13734	2014	117	469	*vanB*	40
UW13735	2015	117	895	*vanB*	41
UW13736	2015	80	315	*vanA*	42
UW13737	2015	117	469	*vanB*	43
UW13738	2015	117	469	*vanB*	44
UW13739	2015	117	469	*vanB*	45
UW13740	2015	192	26	*vanB*	46
UW13741	2015	117	469	*vanB*	47
UW13742	2015	117	469	*vanB*	48
UW13744	2015	780	896	*vanB*	49
UW13745	2015	117	469	*vanB*	50
UW13746	2015	117	469	*vanB*	51
UW13747	2015	117	469	*vanB*	52
UW13748	2015	117	469	*vanB*	53
UW13749	2015	117	469	*vanB*	54
UW13750	2015	192	26	*vanB*	55
UW13751	2015	117	178	*vanB*	56
UW13752	2015	117	469	*vanB*	57
UW13753	2015	117	469	*vanB*	58
UW13754	2015	192	897	*vanB*	59
UW13755	2015	117	469	*vanB*	60
UW13756	2015	117	469	*vanB*	61
UW13757	2015	117	469	*vanB*	62
UW13758	2015	117	469	*vanB*	63
UW13759	2015	192	897	*vanB*	64
UW13760	2015	117	178	*vanB*	65
UW13761	2015	117	469	*vanB*	66
UW13762	2015	117	469	*vanB*	67
UW13763	2015	564	898	*vanB*	68
UW13764	2015	18	253	*vanA*	69
UW13765	2015	117	469	*vanB*	70
UW13766	2015	117	469	*vanB*	71
UW13767	2015	117	178	*vanB*	72
UW13768	2015	117	469	*vanB*	n.d.
UW13769	2015	80	847	*vanB*	73
UW13770	2015	117	469	*vanB*	74
UW13771	2015	117	469	*vanB*	75
UW13772	2015	80	899	*vanA*	76
UW13773	2015	117	469	*vanB*	77
UW13774	2015	17	900	*vanB*	78
UW13775	2015	117	469	*vanB*	n.d.
UW13776	2015	117	469	*vanB*	79
UW13777	2015	117	36	*vanB*	80
UW13778	2015	117	469	*vanB*	81
UW13779	2015	117	469	*vanB*	82
UW13780	2015	117	469	*vanB*	83
UW13781	2015	564	901	*vanB*	84
UW13782	2015	117	469	*vanB*	85
UW13783	2015	117	469	*vanB*	86
UW13784	2015	117	469	*vanB*	87
UW13785	2015	117	469	*vanB*	88
UW13786	2015	117	469	*vanB*	89
UW13787	2015	89	301	*vanA*	90
UW13788	2015	117	469	*vanB*	91
UW13789	2015	117	469	*vanB*	92
UW13790	2015	117	469	*vanB*	93
UW13791	2015	117	469	*vanB*	94
UW13792	2015	117	178	*vanB*	95
UW13793	2015	117	469	*vanB*	96
UW13794	2015	117	469	*vanB*	97
UW13795	2015	117	469	*vanB*	98
UW13796	2015	117	469	*vanB*	99
UW13797	2015	117	24	*vanB*	100
UW13798	2015	117	469	*vanB*	101
UW13799	2015	117	178	*vanB*	102
UW13800	2015	117	178	*vanB*	103
UW13801	2015	117	178	*vanB*	104

The table lists all study isolates (n = 111). In addition, the year of isolation and typing results (sequence type, complex type, and vancomycin resistance type) are listed in detail. In row 6, the patient IDs are also listed, if movement data were available.

^1^ n.d. = no data. For these isolates no patient movement data could be provided.

Four different bioinformatics approaches, namely an SNP-based analysis, cgMLST, feature frequency profiling (FFP), and a maximum common genome (MCG) approach, were used to infer and further to compare outcomes of phylogenetic tree calculations (S1 Fig). By this, the impact of sequence reconstruction on the phylogeny was investigated, since the SNP analysis is based on read alignment to a reference genome and the other methods on *de novo* assembly. All phylogenies were highly consistent for isolates of the predominant genotype ST117/CT469, demonstrating that they were closely related (S1 Fig, blue boxes). The alignment-free FFP approach exhibited lower concordance values when compared to the other three approaches (S1 Fig). Generally, all analyses revealed high values, hence major differences, for isolates that were distantly related. Nevertheless, the comparison indicated a close phylogenetic relationship of the isolates exhibiting the ST117/CT469 genotype, independent of the method of sequence reconstruction.

The three approaches showing 1,707 SNPs, 1,427 cgMLST target alleles, or 1,811 MCG-genes and by this comparable amounts of genetic core approximations. The isolate collection and, in particular, isolates of the predominant genotype, were analyzed in more detail using the data of cgMLST- and SNP-based analyses. By applying reference-based read alignment and variant calling to deduce SNP-based genetic distances, a range of 0 to 802 SNPs was noted for all isolates (S1 Table). By using *de novo* assembled sequences for cgMLST analysis, the genetic distance between study isolates ranged from 0 to 1,105 allele differences (S2 Table). Within the most abundant population, the ST117, the SNP differences raged from 0 to 172. However, and within the ST117/CT469 genotype, SNP-based analyses showed only 0 to 9 SNPs difference between those isolates. A similar result was obtained by cgMLST-based analyses, with allele differences of 0 to 8 between ST117/CT469 isolates only (S2 Fig). The second most abundant ST117 clone, CT178 (n = 7), displayed 0 to 12 SNPs and 0 to 15 allele differences. Isolates not belonging to the most abundant clones showed either genotyping clusters of two to three isolates (n = 12) or were not related to any of the other samples obtained (n = 18). In summary, the comparative results of typing and phylogeny analyses gave evidence for the presence of a dominant clone, which could be validated by further high-resolution investigations of the WGS-based data.

### Data combination, visualization, and examination

After extensive bioinformatics analyses, it was decided to use the results of SNP and cgMLST analyses and to link them with epidemiological information of the corresponding patient group. As only one isolate per patient was obtained, strain IDs could be directly linked to individual patients, their admission, and discharge dates as well as to the respective wards they have been staying at. This information was used to overlay sequencing data in order to infer transmission events and strain dynamics over time. For a total of 105 VRE, patient and epidemiological data were available.

To analyze the impact of patient movements, an interactive tool was designed (S3 Fig). In an interactive session, the tool visualizes where patients were admitted to within the hospital; regarding further aspects of potential patient-to-patient connections, the ward the patient was admitted to when the VRE isolate was obtained, in combination with the phylogenetic data, was determined, thus allowing extraction of information important for downstream analyses or intervention measures (S1 Movie). The designed tool is provided as a ZIP file (S1 File).

The interactive tool visualized that the predominant clone ST117/CT469 was present in all clinical wards investigated and over the entire screening period (S3 Fig). A large number of potential patient contacts was identified. We selected particular isolate groups in the context of time and genetic relationship and analyzed those in more detail. One example group (S3B Fig) consisted of the dominant clone ST117/CT469, isolated from five patients (patient IDs: 43, 47, 48, 50, 51; see Table 1). By manually monitoring the patients' movement data using the interactive functions of the instrument, a possible transmission chain could be described, in which two patients shared a room at the same time or at different times (Table 1). For comparison and as a second example, patient contacts of the clone with the ST117/CT178 genotype were also reviewed. The patients were admitted to six different hospital wards and 22 rooms. Five of the seven patients had been traced back to the fact that they were admitted to the same patient rooms and therefore could potentially have come into contact with each other.

A potential transmission of the clones across different wards could be predicted based on the room occupancy of certain patients. Further, by revisiting the cgMLST- and SNP-based analyses, the corresponding isolates proved to be closely related for both predominant clones. The possibility of patients of both described clones to form a transmission chain will be further investigated by valid mathematical considerations in the following section.

### Assessment of “infectious-contact time” by patient contact network modeling

**Fig 3:**
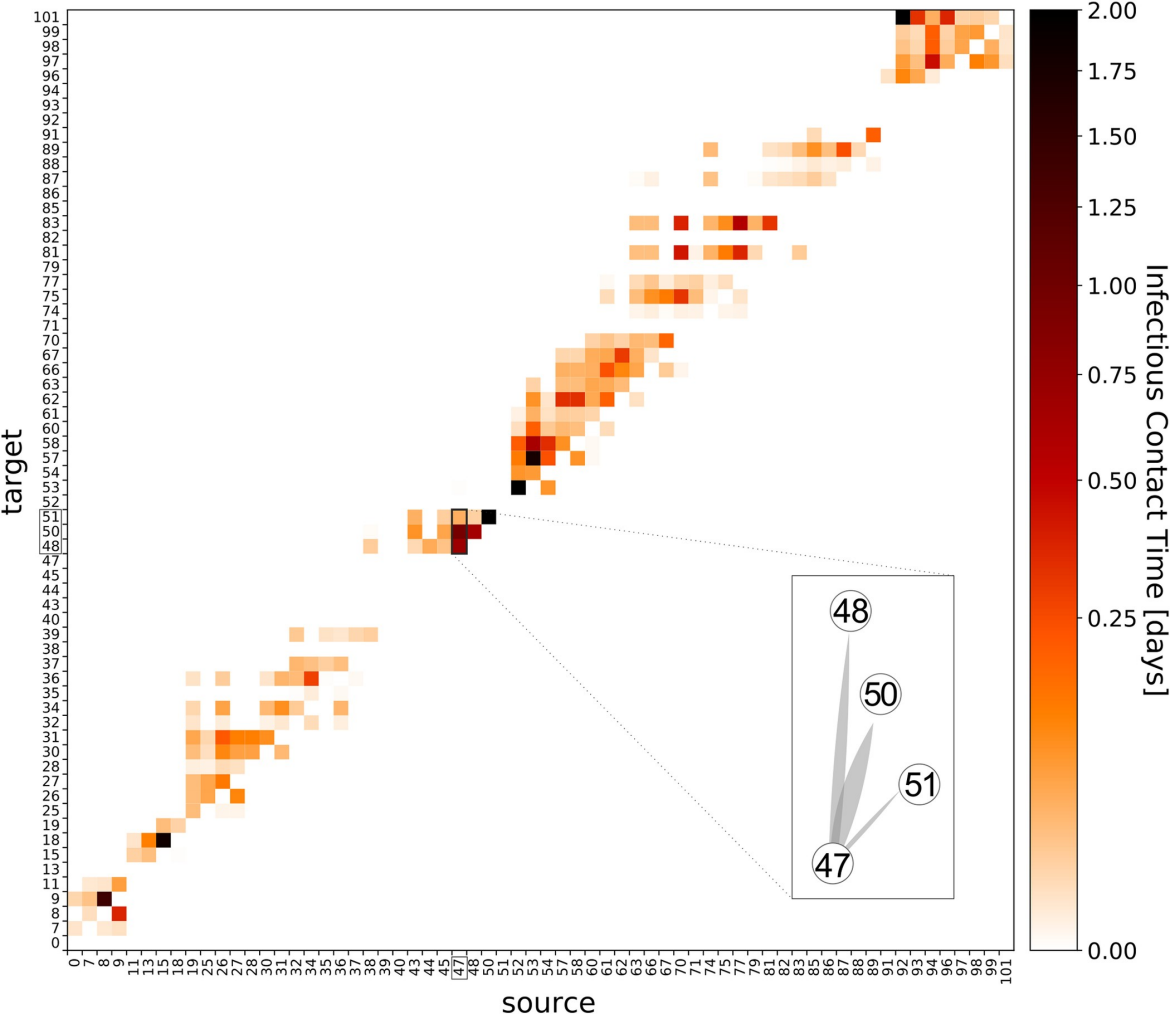
Heatmap of the „infectious-contact time” for the patient group carrying the ST117/CT469 clone. The value was defined and calculated on basis of spatial and temporal proximity between patients. All patients are treated as both potential ‘source’ and ‘target’ of VRE colonization and are therefore represented by patient IDs (compare with Table 1) on the diagram axes. The “infectious-contact time” value is high (up to 2; dark color) when two patients were close in time and in space and low (or zero, light color) when a potential contact of two patients was far or unlikely (compared with scale bar). The matrix shows “infectious-contact times” between source patients (columns) and target patients (rows) in a logarithmic color scale. Zero rows or columns indicate patients who can likely be excluded as target or source of colonization. By taking the epidemiological data into consideration, there are patients being a potential multiple source for or multiple targets of other patients; as example a zoom was highlighted showing the connection of patient 47 as source for target patients 48, 50 and 51 (see Fig 4).

To compare the previous qualitative analysis with a rigorous quantitative method, the “infectious-contact time” was computed for the patients colonized by clone ST117/CT469 (for a detailed explanation of the “infectious-contact time”, see S1 Appendix). For this, we regarded all patients from this cohort as both potential sources and targets of VRE transmissions. In the following, patients were identified with a patient ID (please compare with Table 1 for the patient-isolate key), and for each ST117/CT469 isolate of the following described groups, the genetic distances were visualized (S2 Fig). The “infectious-contact time” between source patients and target patients was visualized in Fig 3. Non-zero values indicate that a transmission between these patients could have occurred according to our assumptions of patient incubation time and room recovery time (see S1 Appendix). As described in the Method section, the “infectious-contact time” is high if source and target were in spatial and temporal proximity during the target's strain acquisition period, revealing and excluding potential contacts between patients leading to transmission.

**Fig 4:**
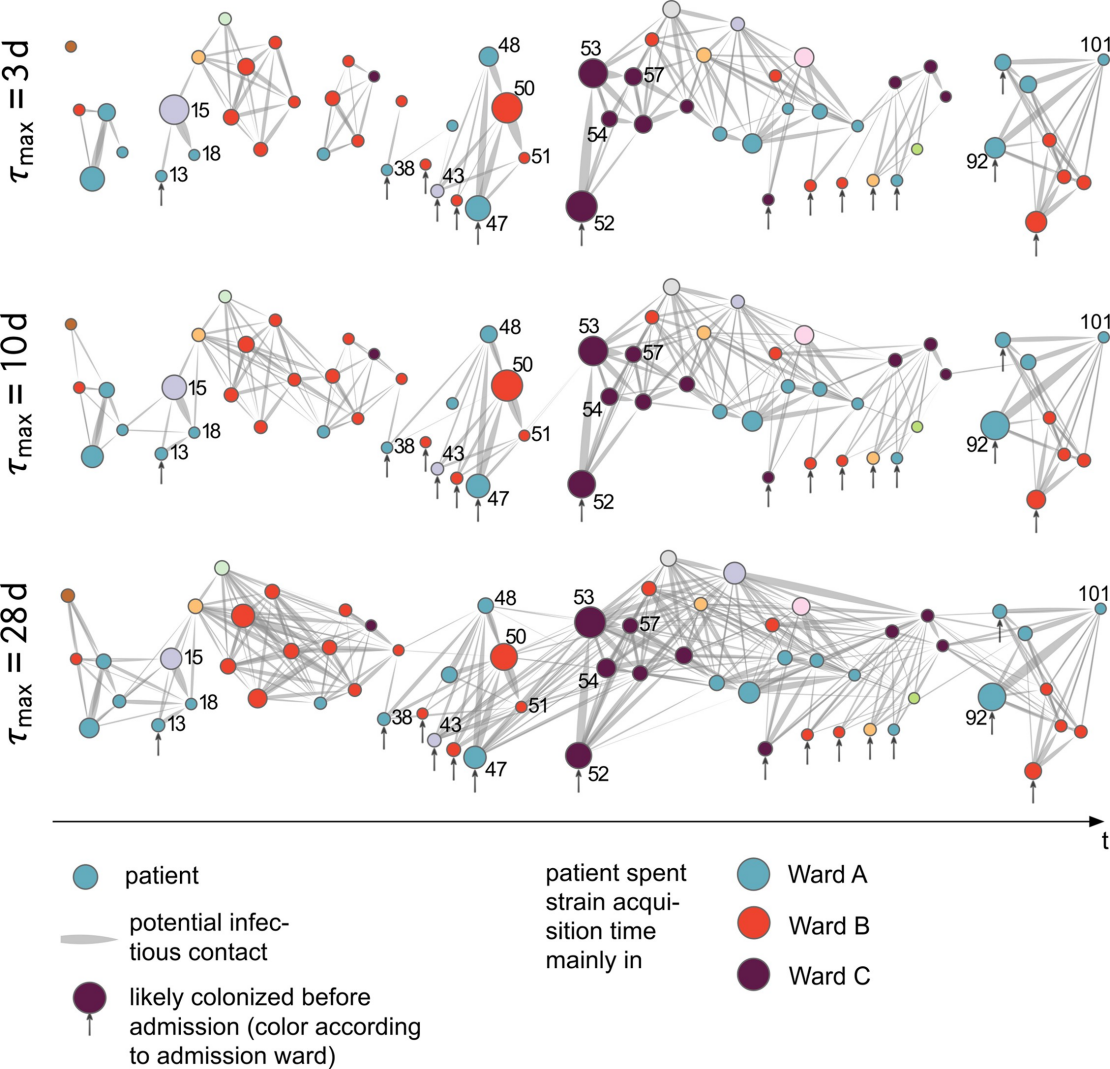
Putative transmission network of patients carrying the predominant ST117/CT469 clone. Patients are represented by nodes (colored disks) and by patient IDs (compare with Table 1), and the infectious-contact time between a target-source pair (compare with Fig 3) is shown as a weighted, directed link (wedged lines). The node size is scaled by the aggregated amount of time they may have been responsible for an infection of other patients. Due to the large heterogeneity in “infectious-contact time” between pairs of patients, we scaled the link width for visualization, by potentiating the “infectious-contact time” with 0.3, underemphasizing large contact times to facilitate the identification of clusters. Patients who most likely carried the strain at admission are marked with a vertical arrow and colored by their admission ward. The color of the other nodes refers to the ward they spent most of their strain acquisition time in. The horizontal position of nodes roughly corresponds to increasing integer values, ordered by the time of their first positive VRE test (i.e. the time-scale is not linear). Different recovery times (τmax) were applied, resulting in different contact network complexity. A) recovery time was set to 3 d; B) recovery time was set to 10 d; C) recovery time was set to 28 d.

Based on this “infectious-contact time” matrix, representing potential patient contacts, we constructed an “infectious-contact network”. The network both visualizes the contacts in a spatio-temporal manner and allows transmission modeling by varying particular parameters. An example is the room recovery time, which represents the time a room may still be contaminated by VRE after a patient´s discharge, based on cleaning and disinfection. The results are shown in Fig 4, where nodes (circles) represent patients and potential transmissions are shown as weighted, directed links (wedged lines). Nodes were colored according to the ward the patients spent most of their strain acquisition time in. Fifteen nodes (~23%), marked with vertical arrows, represent patients who most likely were colonized before they were admitted to the hospital (i.e., their positive samples were taken within 2 days of being admitted). These “index patients” were colored by their ward at admission (Fig 4). Patients 13, 38, 43, 52, and 92 of this group were the first positively tested patients in an otherwise disconnected component of the network (Fig 4A). Furthermore, four of the ten highest “infectious-contact times” were associated with source nodes of this patient group (pairs 47–50, 47–48, 52–53, and 92–101) (Fig 3).

Using an adjusted room recovery time of 10 d supported these findings and closed some of the transmission gaps between formerly disconnected transmission components (Fig 4B). To dismiss the possibility that the index patients imported the clone into the hospital, a rather short incubation time of <1 d is required, and a room recovery time of up to 28 d must be assumed to close the last transmission event gaps between the formerly disconnected components of the network (Fig 4C). Nevertheless, the former index patients would still be associated with unusually high “infectious-contact times” towards other patients. Longer chains of possible transmission events can be clearly observed between patients contained in the three mainly screened wards (wards A, ward B, and ward C; Fig 4). Besides intra-ward, several strong inter-ward “infectious-contact times” were observed. The main inter-ward connections were between wards A and B (both directions) as well as from ward C to ward A. Two potential strong transmission chains between four patients were revealed, supported by molecular data, each associated with very high “infectious-contact times” compared to the rest of the network: 47-48-50-51 and 52-53-54-57 (Fig 4).

In a second analysis, the “infectious-contact time” was computed between all seven patients colonized by the second abundant clone ST117/CT178. Despite the fact, that these patients were in close spatial proximity (mostly in the same ward), at least four of them were most likely already colonized during admission. Furthermore, there was a 2-month gap between the discharge of the third colonized patient and the admission of the fourth patient colonized with this clone. Merely two of those patients can be associated with colonizations obtained within the hospital.

In summary, the “infectious-contact time” analysis of patient groups carrying the most prevalent clones showed the importance of reassessing patient contacts in a spatio-temporal context to define a high or low risk for transmission. Further, visualizing potential contact-times as an “infectious-contact network”, is an accessible method to easily infer potential transmission chains and to demonstrate the influence of two particular parameters: room recovery time, which can be directly linked to hygienic measures, and patient incubation time, from which the possible influx of pathogens into the hospital can be inferred.

## Discussion

To understand the emergence and to prevent the further spread of multi-drug-resistant pathogens, it is important to monitor the prevalence and transmission of certain strain types in clinical settings. In this study, we retrospectively analyzed a setting in which high-risk patients from a tertiary care hospital in Germany were routinely screened for VRE colonization. We investigated the performance of different bioinformatics analyses for WGS-based typing. We further combined WGS-based results with clinical and epidemiological data and created a patient-based contact network to retrospectively assess the benefits of combining all available data sources, identifying transmission hotspots and improving direct measures of infection control to the most influential sites for an effective intervention.

### Hygiene measures

This study focused on VRE-carriage as the prerequisite of a subsequent VRE infection. A high screening rate was achieved by screening on admission and upon discharge, but foremost by regular weekly screening dates for all patients on the respective wards. Patients with short duration of stay and patients treated for reasons other than hematological disorders might not have been screened. In patients of the present study, mostly suffering from malignant hematological disorders, high-resolution typing revealed a high degree of clonality suggesting a local transmission network. The retrospective analysis therefore implies that the infection prevention bundle routinely employed was not able to prevent VRE transmission. This bundle was composed of standard hygiene procedures complemented by rigorous screening and consecutive single room placement of patients combined with contact isolation measures, according to the current recommendations of the commission for hospital hygiene and infection prevention (KRINKO) [56]. Hand hygiene was routinely trained, and the consumption of alcohol rubs was assessed; however, compliance to indications of hand hygiene was not measured. In light of the clonality of strains and the transmission events elucidated herein, additional and/or alternative infection control measures need to be focused on in the near future. They would include antibiotic stewardship, patient empowerment, avoidance of understaffing, and active hand hygiene compliance monitoring. Furthermore, strict control and, possibly, further broadening of surface cleaning and disinfection procedures need to be considered. The appropriateness of single room placement of VRE-patients itself is a matter of debate for economic and patient safety issues, especially in Germany [57]. The relative economic burden imposed by single room isolation in the hematological-oncological setting is very high if the number of putative infections observed in this study is taken into account. In the setting described herein, only in two of the patients, VRE were isolated from clinically relevant material.

### Molecular analyses and bioinformatics

Most of the VRE isolates belonged to prominent German hospital-associated *E*. *faecium* lineages such as ST117, ST80, ST192, or ST203; almost all of them exhibited *vanB*-type vancomycin resistance, which recently emerged in German clinics [11]. Also as demonstrated by Hammerum *et al*., Danish VRE analyzed from 2005 to 2015 revealed genotypes such as ST80 (CT14), ST117 (CT24), and ST203 (CT859) [58]. Our study proved genotype ST117/CT469 as the predominant clone that was occasionally detected also in other countries such as Luxembourg and the Netherlands (www.cgMLST.org) and is known to be distributed across Germany [59].

In Australia, a study was conducted to obtain insights into the general VRE population of different hospitals. The researchers observed ST796 as the dominating lineage of the investigated region [60]. Hospital screening settings from Denmark observed the spread of several clones, including ST117, ST80, ST78, and ST192, and could further deduce VRE transmissions between patients, wards, and even hospitals. This was performed by pairwise SNP comparisons merged with epidemiological data [61]. In 2019, Liese *et al*. focused on VRE colonization within a hospital in Southwest Germany and also revealed a high prevalence of *vanB* type VRE and of the same STs as observed in our study [62]. All three mentioned studies demonstrated the suitability of WGS in combination with epidemiological data for outbreak analyses; however, without applying cgMLST analyses, the results cannot be used for high-resolution inter-study comparisons.

In the present study, WGS-based analyses served as a basis for different phylogenetic calculations. The three bioinformatics methods that were based on the reduction of the genetic entity to a core size (genes = MCG, alleles of genes = cgMLST, SNP-based) were generally comparable, in particular, a good concordance for highly related isolates was observed. Similar results have been achieved by comparative analyses between SNP-based and cgMLST-based phylogenies of *Listeria monocytogenes* by Henri *et al*. [42]. Additionally, we applied and compared the FFP approach, which is based on the complete genetic information, which seemed to generate reliable clustering results for *E*. *faecium*. Considering all four approaches, we observed a generally lower concordance for distantly related isolates. This fact could be explained by the different methods for sequence reconstruction and the differing core-size approximation. For example, the SNP analyses are based on read-alignment and therefore dependent on a suitable reference genome, whereas the other analyses used *de novo* assembled sequences. While cgMLST uses a defined core genome reference, the MCG approach calculates an *ad hoc* core genome of the included sequence collection, also including accessory elements such as the FFP method. Other studies relied on SNP-based analyses only by using a proper and highly related reference genome to enhance the power of discrimination by this approach [60]. While these analyses provide results in high resolution, they are lacking comparability and transferability to other settings [41]. In our study, especially the combination of SNP-based and cgMLST-based results produced valid and comparable data. Notably, a higher resolution was revealed by the cgMLST approach, e.g., for the group with ST117/CT178, which in turn highlights the imperative necessity of choosing the best reference genome for SNP-based analyses [63]. For *E*. *faecium*, in both multi-clonal outbreak and population analyses, it is often a trade-off to use a suitable reference sequence for the entire collection instead of using the best reference for each sub-set of strains [60]. Also, applying filter processes to exclude putative genetic entries by recombination events, could affect the results of SNP-based analyses [39]. For reasons of general concordance, reproducibility, and inter-lab comparability, this again emphasizes the advantages of applying cgMLST analyses in *E*. *faecium* outbreak situations, especially for poly-clonal settings.

### “Infectious-contact” network-based approach

Nowadays, the usage of WGS analyses is regarded as a standard repertoire for detailed population studies and outbreak analyses [35–37, 61]. The particular focus on patients sharing the same room and declaring those as contacts for putative transmission events was also recently described in other studies [35, 64]. In contrast to these studies, we further included the spreading potential of contaminated patient rooms as a second parameter, since this was identified as an important risk factor for VRE transmission [27]. We thus constructed potential “infectious-contact networks” for two distinct sub-groups in high resolution, partitioned by their colonizing VRE clone. We showed that the predominant ST117/CT469 clone was most likely imported into the hospital several times, even though the isolates were highly identical. A possible explanation of this phenomenon could be the re-admission of patients into the hospital several times or the admission to other hospitals or healthcare facilities before. Unfortunately, no such data were available for this study. Each time the clone was imported, it caused a small-scale transmission cascade within wards and between wards, mainly revealing spreading events between three major wards. The impact of hospital staff in the model could not directly be covered, but we assume a close contact between patients and hospital staff; in this sense, inter-ward transmission was also caused by patient movements.

The “infectious-contact network” analysis of patients carrying the ST117/CT178 clone indicated that multiple import events are responsible for the prevalence of this strain. Only two of the seven patients had obtained the strain from other patients. Only if modeling parameter values for incubation and room decontamination times were changed to theoretical values from *in-vitro* studies, the model prediction of VRE influx into the hospital broke down. A recovery time of 28 d would imply that a room would have had to be cleaned at least 28 times until it could be considered decontaminated or that its contained clones were spread to unmonitored areas from which further spread would have been possible. The adjustments of the recovery time also give hints to the complexity of possible transmission routes within the hospital and can exemplarily demonstrate how different clones of the same species could be transmitted in general. Transmission events were inferred within groups carrying an identical clone only. Furthermore, data on all (not only positively tested) patients within the hospital should be used to compare the movements of colonized patients to those not carrying VRE, possibly revealing intra-hospital pathways of spreading [29].

### Limitations

Our study has several limitations. The study design is retrospective and observational. Thus, inclusion and exclusion criteria were not defined in advance; adherence to infection prevention and control policy was not systematically assessed. This includes the implementation of isolation procedures, the compliance to hand hygiene, as well as environmental cleaning procedures. For reasons of patient compliance and ease of use, VRE screening was conducted by perianal and not by rectal swabs. There is indirect evidence that carriage rates are underestimated if perianal swabs are used [65]. Further, we have no information about possible patient movements between different hospitals in the same region and no data about the VRE situation in these clinics; the possible influence of regional healthcare networks has been documented [66]. The model accounts for the movements of hospital staff only indirectly, and therefore, their influence on transmission or persistence could not be included [28, 29]. Future analyses should include staff movements. Another limitation is that we only used one isolate per patient and that the time of positivity and the length of hospital stay varied among patients.

## Conclusions

In this study, we present an effective strategy that comprehensively combined different approaches and disciplines for successful interventions in healthcare-associated infection management and the development of anti-infectious strategies. Additionally, the herein developed “infectious-contact modeling” approach is a promising tool for controlling infection prevention bundles. We further highlight the importance of interdisciplinary cooperation to mutually validate results and demonstrate step-by-step how each discipline provided an added value to unravel the complex routes of VRE transmission.

## Supporting information

S1 Table SNP-distances. This table contains the supporting information about SNP-distances of 111 isolates of the entire study collection from SNP-based read-alignment and variant-calling approach against reference genome *E*. *faecium* 64/3 (NZ_CP012522.1).
(XLSX)

S2 Table Allele-distances. This table contains the supporting information about allele-distances of 111 isolates of the entire study collection from core genome MLST-based analyses using the SeqSphere^+^ software.
(XLSX)

S1 Movie Demonstration of an interactive session of the developed tool for data visualization. The cursor moves over a hospital ward on the right side and all bars corresponding to the individual patients on the particular ward are highlighted. Furthermore, the cursor moves over a single day on the patient bar and all patients located in the same hospital room at any time will be highlighted and visually connected. Additionally, to obtain patient data and genotyping information, a phylogenetic tree was added to visualize the information of strain relationships.
(MOV)

S1 File Developed tool for data visualization. The folder includes all necessary files and documents to run the developed tool.
(ZIP)

S1 Fig Visualization of the comparison of the phylogenetic concordance. To visualize the concordance of the four applied tree building methods, heatmaps were created which were calculated based on the particular absolute patristic distances for each isolate obtained by two different approaches A) the SNP-based (read-alignment and variant calling) approach and B) cgMLST analysis (based on *de novo* assembly), C) maximum common genome (based on *de novo* assembly) or D) feature frequency profiling (FFP; based on *de novo* assembly)—based phylogenetic trees. The left side of the heatmap depicts the phylogenetic tree which was used as reference for the comparison (A/B/C). On the top of the map are the phylogenetic trees for B) cgMLST, C) maximum common genome and D) FFP-based analyses. The heatmap color-codes the absolute difference of patristic distances of isolate pairs obtained by the respective phylogenetic analyses. Small differences in distances are shown in blue. Large differences in distances are shown in orange. The median of all values was calculated to lower the effect of logarithmic data distribution on the visualization and used as the mean value of the color scale, which is shown in gray (“13”).
(PDF)

S2 Fig Visualization of read-alignment and variant calling determination of SNPs and *de novo* assembly-based cgMLST-allele differences between the isolates belonging to the predominant clone with genotype ST117/CT469. The number of differences is represented by different shades of grey (see legend). Left, a phylogenetic tree on basis of the SNP analyses is shown. The left part of the heatmap displays the SNP differences and the right part the respective allele differences for each isolate pair. The diagonal black boxes show no value, but denote the dividing line between SNP- and allele differences. Visualization was realized using iTOL.
(PDF)

S3 Fig Screenshot of the developed tool for patient data visualization. A) The left side of the figure depicts the SNP-based phylogenetic tree of 105 VRE from the routine screening and for which patient and epidemiological data were available. Results of the genotyping were added for each isolate: the colored background refers to the sequence type. The enclosing lines delineate the particular complex types. The x-axis shows the timeline for the screening period. Isolates of the phylogenetic tree are indicated for all patients. The length of the isolate bar corresponds to the time each patient was investigated. On the right site, hospital wards where patients were admitted to are listed and color-coded (for personal privacy, we anonymized the wards for this figure). B) The dotted box highlights a patient group that was selected for manual analyses in detail.
(PDF)

S1 Appendix Inferring and defining the “infectious-contact time” based on patient-based data. Here, we aim at computing the total amount of time in which a patient *j* can be responsible for the infection of a patient *i* and introduce two tunable parameters which let us control the influence of the hospital structure as well as the cleaning procedures more accurately. Details regarding parameters and assumptions are described in this appendix.
(DOCX)

## Abbreviations

cgMLST: core genome multilocus sequence typing
CT: complex type
FFP: feature frequency profiling
MCG: maximum common genome
MLST: genome multilocus sequence typing
PFGE: pulsed-field gel electrophoresis
SNP: single nucleotide polymorphism
ST: sequence type
VRE: vancomycin-resistant *Enterococcus faecium*
WGS: whole-genome sequencing
