# Supplementary material for: Comprehensive integrated NGS-based surveillance and contact-network modeling unravels transmission dynamics of vancomycin-resistant enterococci in a high-risk population within a tertiary care hospital
Source: PLoS One. 2020 Jun 24;15(6):e0235160. doi: 10.1371/journal.pone.0235160 (PMC7314025; doi:10.1371/journal.pone.0235160)
Supplement: S1 File — The folder includes all necessary files and documents to run the developed tool. (ZIP) [file pone.0235160.s004.zip › VRE-line-chart-master/index.html]

|  |  |
| --- | --- |
| Patient |  |
| Isolate |  |
| Date/time of isolation |  |
| Ward at isolation |  |
| Ward |  |
| Room |  |
| Bed |  |
