# Supplementary figures and images for: Comprehensive integrated NGS-based surveillance and contact-network modeling unravels transmission dynamics of vancomycin-resistant enterococci in a high-risk population within a tertiary care hospital
Source: PLoS One. 2020 Jun 24;15(6):e0235160. doi: 10.1371/journal.pone.0235160 (PMC7314025; doi:10.1371/journal.pone.0235160)

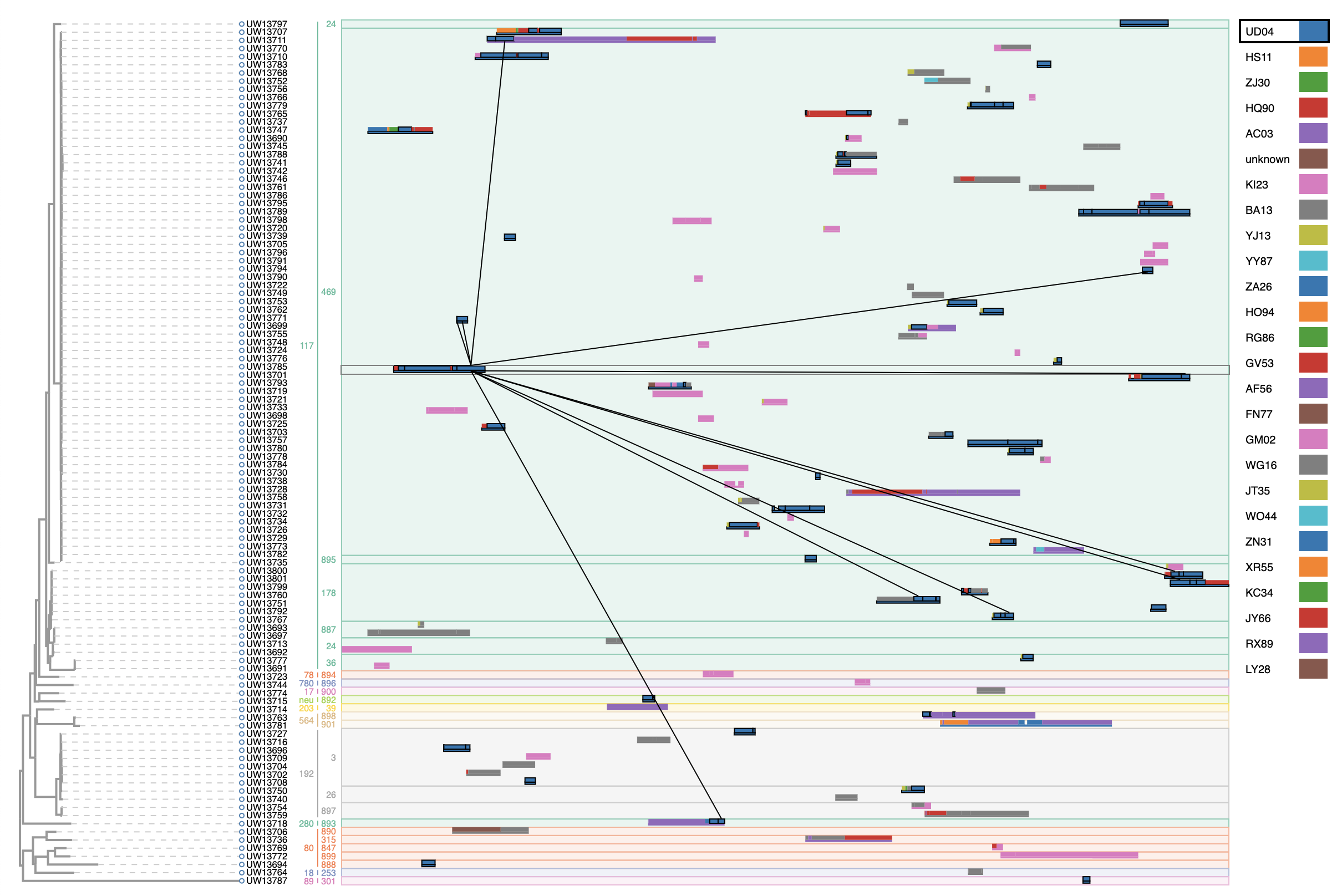

Supplement: S1 File — The folder includes all necessary files and documents to run the developed tool. (ZIP) [file pone.0235160.s004.zip › VRE-line-chart-master/img/example.png]

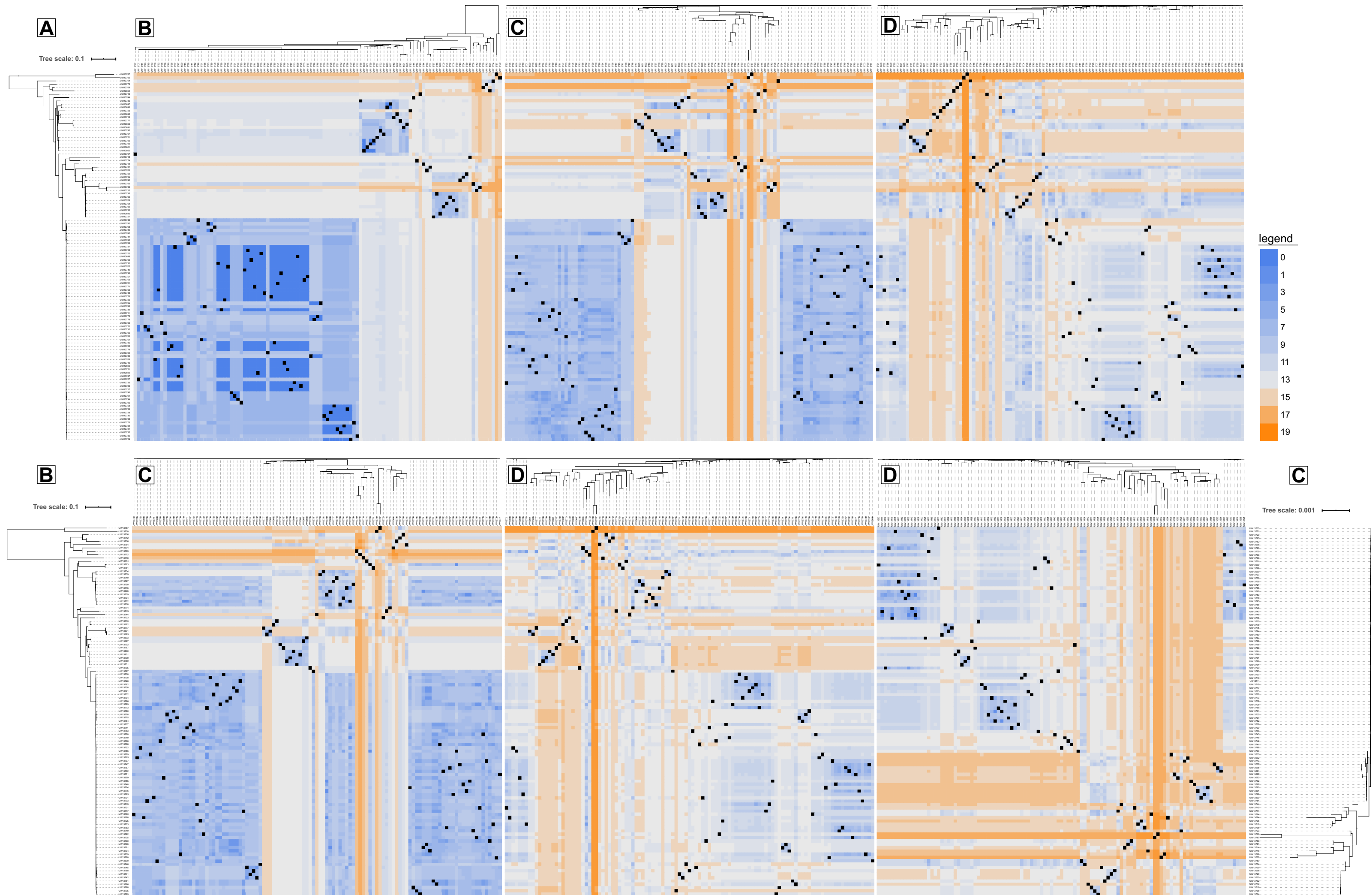

Supplement: S1 Fig — To visualize the concordance of the four applied tree building methods, heatmaps were created which were calculated based on the particular absolute patristic distances for each isolate obtained by two different approaches A) the SNP-based (read-alignment and variant calling) approach and B) cgMLST analysis (based on de novo assembly), C) maximum common genome (based on de novo assembly) or D) feature frequency profiling (FFP; based on de novo assembly)—based phylogenetic trees. The left side of the heatmap depicts the phylogenetic tree which was used as reference for the comparison (A/B/C). On the top of the map are the phylogenetic trees for B) cgMLST, C) maximum common genome and D) FFP-based analyses. The heatmap color-codes the absolute difference of patristic distances of isolate pairs obtained by the respective phylogenetic analyses. Small differences in distances are shown in blue. Large differences in distances are shown in orange. The median of all values was calculated to lower the effect of logarithmic data distribution on the visualization and used as the mean value of the color scale, which is shown in gray (“13”). (PDF) [file pone.0235160.s005.pdf]

Tree scale: 0.01

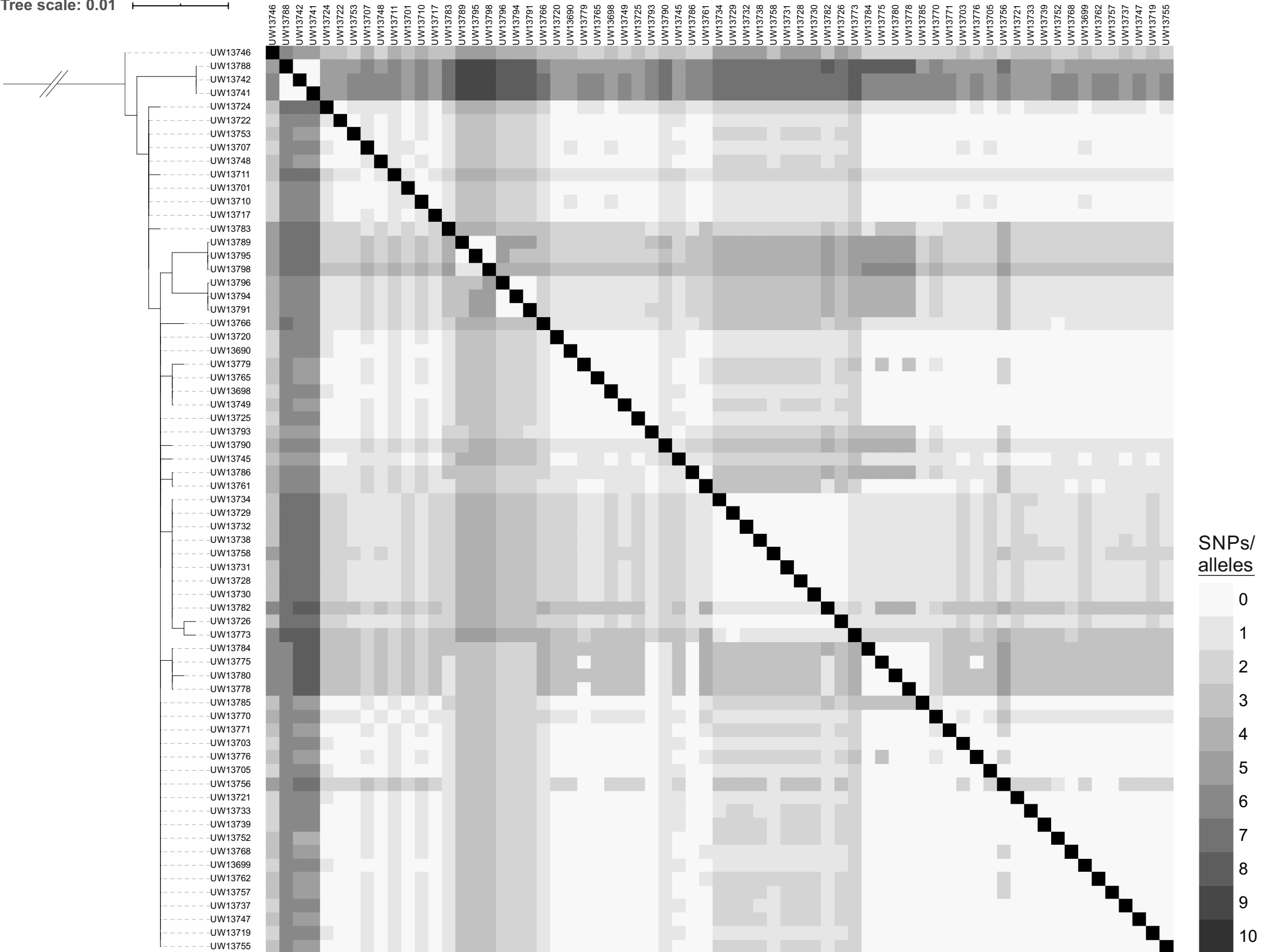

Supplement: S2 Fig — The number of differences is represented by different shades of grey (see legend). Left, a phylogenetic tree on basis of the SNP analyses is shown. The left part of the heatmap displays the SNP differences and the right part the respective allele differences for each isolate pair. The diagonal black boxes show no value, but denote the dividing line between SNP- and allele differences. Visualization was realized using iTOL. (PDF) [file pone.0235160.s006.pdf]

A

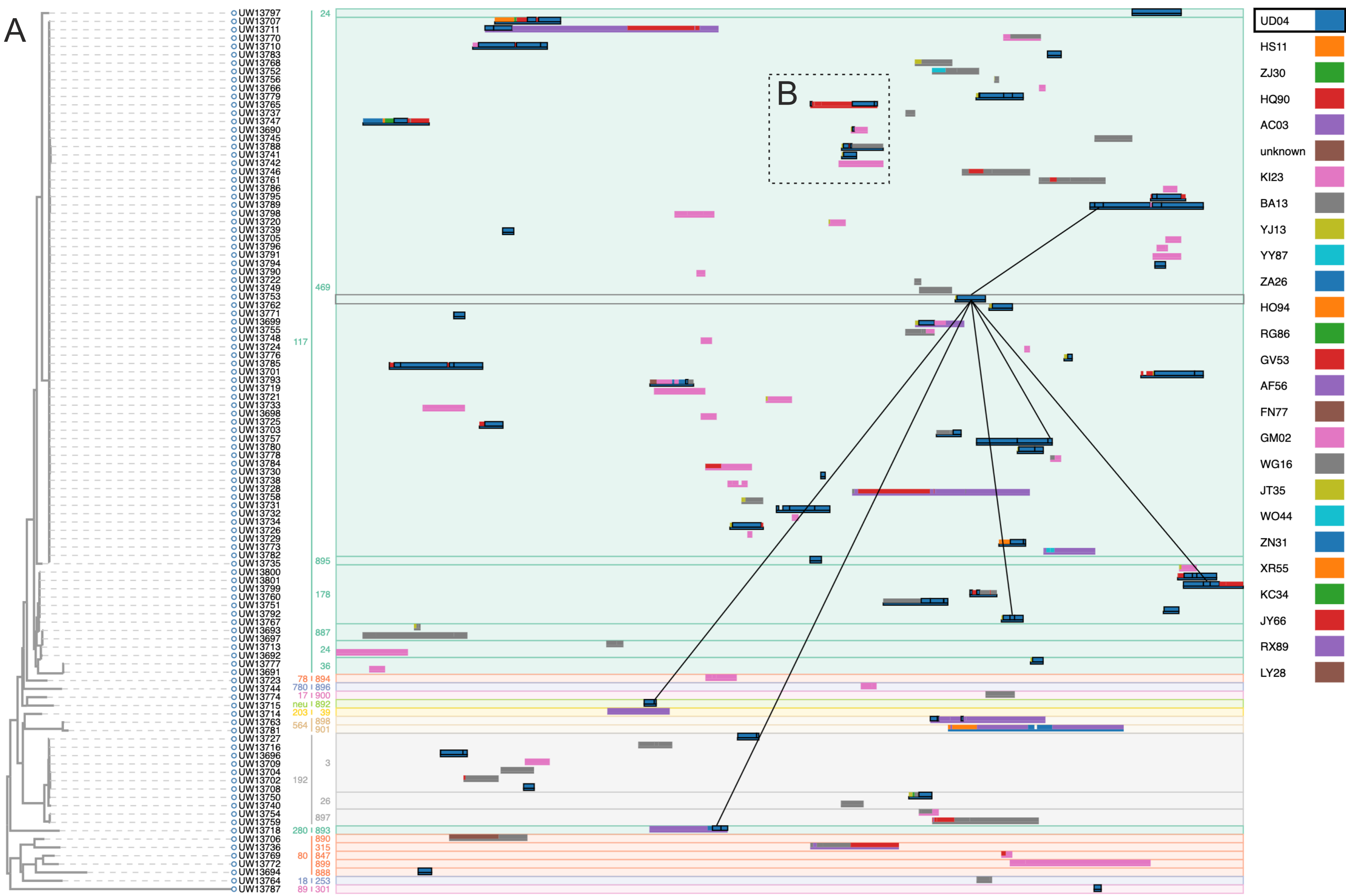

Supplement: S3 Fig — A) The left side of the figure depicts the SNP-based phylogenetic tree of 105 VRE from the routine screening and for which patient and epidemiological data were available. Results of the genotyping were added for each isolate: the colored background refers to the sequence type. The enclosing lines delineate the particular complex types. The x-axis shows the timeline for the screening period. Isolates of the phylogenetic tree are indicated for all patients. The length of the isolate bar corresponds to the time each patient was investigated. On the right site, hospital wards where patients were admitted to are listed and color-coded (for personal privacy, we anonymized the wards for this figure). B) The dotted box highlights a patient group that was selected for manual analyses in detail. (PDF) [file pone.0235160.s007.pdf]
