# Supplementary material for: Comprehensive integrated NGS-based surveillance and contact-network modeling unravels transmission dynamics of vancomycin-resistant enterococci in a high-risk population within a tertiary care hospital
Source: PLoS One. 2020 Jun 24;15(6):e0235160. doi: 10.1371/journal.pone.0235160 (PMC7314025; doi:10.1371/journal.pone.0235160)
Supplement: S1 Appendix — Here, we aim at computing the total amount of time in which a patient j can be responsible for the infection of a patient i and introduce two tunable parameters which let us control the influence of the hospital structure as well as the cleaning procedures more accurately. Details regarding parameters and assumptions are described in this appendix. (DOCX) [file pone.0235160.s008.docx]

**S1 Appendix. Inferring and defining the “infectious-contact time” based on patient-based data.** Here, we aim at computing the total amount of time in which a patient $j$ can be responsible for the infection of a patient $i$ and introduce two tunable parameters which let us control the influence of the hospital structure as well as the cleaning procedures more accurately. Details regarding parameters and assumptions are described in this appendix.

In this study, possible patient-to-patient transmission pathways are inferred from patient movement data. In recent studies face-to-face contacts between patients, between staff, and between patients and staff with were recorded with a high temporal resolution using radio frequency ID chips to find transmission pathways for the spread of diseases like influenza [1, 2]. However, most hospitals do not provide monitoring on such a detailed scale. Furthermore, such data ignores transmission paths where surfaces in hospital rooms act as vectors. It is known that VRE are constantly shed by colonized people and easily survive on surfaces in hospital rooms on a timescale of months, allegedly making the room of a patient a strong vector for transmission to other patients. Such important scenarios are typically ignored when only analyzing person-to-person contacts. There is further evidence that cleaning personnel act as a vector to transmit VRE colonies between rooms and that health care staff acts as vectors to transmit VRE between patients; it has been shown that the application of improved cleaning methods therefore reduces the number of spread. Modern hospitals typically collect data on patient movements but do not collect the movements of any hospital staff. Therefore, pathways have to be inferred implicitly by considering that colonies may spread between rooms due to the action of hospital personnel. To this end, a bipartite perspective will be chosen here, where both patients and hospital rooms can be colonized by strains, with unrecorded staff movements acting as an implicit distribution system. A similar approach has been chosen in a recent study investigating an outbreak in a hospital in Southern Germany where contacts between patients have been inferred by considering their movement data, potential spreading between rooms, and the influence of infections acquired from surfaces long after an infected patient left a room. Here, we aim at computing the total amount of time in which a patient $j$ can be responsible for the infection of a patient $i$ and introduce two tunable parameters which let us control the influence of the hospital structure as well as the cleaning procedures more accurately. The inference method is based on a few key ingredients.

1. Patients are located in rooms. When patients are colonized by a VRE strain, they can be responsible for the colonization of other patients later on. As there exists no information about direct person-to-person contacts, infections have to be modeled as being transmitted via rooms, i.e. a room will be regarded as infectious as soon as it hosts an infected patient. We denote rooms with lower case Greek letters $\alpha,\beta$ and patients with lower case latin letters $i,j$. A function $\alpha_{i}(t)$ returns the room the patient $i$ is in at time $t$.
2. It is assumed that a hospital room acts as a vector to transmit a strain between patients. Less clear is the influence of the overall hospital organization. Hospitals are usually organized in a hierarchical manner: A hospital may be divided into several buildings, which host several wards, which are again divided in rooms, defining a hierarchical tree of structural organization. In order to control the influence of this organization on VRE spreading between rooms, we define two rooms to be of a certain "distance" $D$ given by their position in this hierarchical tree. The distance between a single room $\alpha$ and itself is $D(\alpha,\alpha)=0$. The distance between two rooms $\alpha$ and $\beta$ in the same ward is $D(\alpha,\beta)=1$, the distance between two rooms which are not in the same ward but in the same building is $D(\alpha,\beta)=2$ and so on. *Footnote: Mathematically speaking, the distance of two rooms is given by the minimum number of steps necessary to reach their lowest common ancestor in the hierarchical tree.* Transmissions between rooms are implicitly assumed by introducing a distance-dependent strain flux between a patient staying in room $\alpha$ and a patient in room $\beta$. This flux is defined as $F_{\alpha\beta}=\xi^{D(\alpha,\beta)}$. Here, we use the parameter $0\leq\xi\leq1$ to control the influence of the hospital structure on possible infectious contact-time. The potential flux from a patient to another patient is proportional to $F=1$ if they stayed in the same room, $F=\xi$ if they stayed in the same ward but not in the same room and so forth. *Footnote: Using this form of influence exponentially decaying with distance is an established way to ensure that the total flux between an exponentially growing number of partners with distance* $D$ *is of similar order* [3, 4]*.* Using this flux, $\xi=0$ corresponds to a situation where patient $i$ can only be responsible for a colonization of patient $j$ if they stayed in the same room and $\xi=1$ corresponds to a situation where the hospital structure does not matter at all: Every room can be responsible for a colonization in every other room. This would, for instance, describe a real-world situation in which cleaning and health care staff quickly traverse between any part of the hospital and thus rapidly spread colonies to every room. Varying the value of $\xi$ will be used to identify how strongly the influence of mixing has to be assumed in order to establish possible infection pathways between patients colonized with similar strains. In the recently published study about an outbreak in Southern Germany a similar approach was taken where the flux of strains between patients in rooms of the same ward was set to be a third as strong as the flux of strains between patients in the same room, which would correspond to $\xi=1/3$ here^10^. In contrast to this study, flux between rooms of greater hierarchical distance is still possible in our method.
3. Patients can be either colonized or susceptible for a colonization, quantified by the infection function $I_{i}(t)$. A value of $I_{i}(t)=1$ means that patient $i$ is colonized with a strain and potentially infectious to other patients. It also implies that they cannot be infected with a different strain anymore such that their susceptibility status is $S_{i}\left( t \right)=0$. In contrast, $I_{i}(t)=0$ implies that the patient is not infected and susceptible for colonization. In general, $S_{i}\left( t \right)=1-I_{i}(t)$ if the patient is currently in the hospital and $S_{i}(t)=I_{i}(t)=0$ if they are not. For each patient we know the date of their positive VRE test. Since the test type was rectal swaps, a colonization may take up to two days to be detectable, we assume$I_{i}(t)=1$ if $t>t_{i}^{test}-2d$. Furthermore, patients were tested on a weekly basis, there is strong reason to believe that the infection must have been acquired between $t=t_{i}^{test}-9d$ and $t=t_{i}^{test}-2d$. We model this by assuming that the infection status increases linearly over time from $I=0$ at $t=\max(t_{i}^{test}-9d,t_{i}^{adm})$ to $I=1$ at $t=t_{i}^{test}-2d$ where$t_{i}^{adm}$ is the hospital admission time of patient $i$. The weekly test also implies that this time interval is the only time interval which has to be considered for patient $i$ to have acquired a strain.
4. A room $\beta$ is considered to be infectious with the strain of patient $j$ as soon as patient $j$ is in the room. The room infection status with respect to patient $j$ is denoted by the function $Y_{\beta j}(t)$. When patient $j$ is in room $\beta$ at time $t$, then $Y_{\beta j}(t)=I_{j}(t)$. Once a room has been infected, it can be considered to carry the strain of patient $j$ for a longer time, even if the patient is not in the room anymore. Cleaning rooms decreases the chance of colonies surviving on surfaces and therefore decreases the probability that a patient is responsible for the colonization of another patient when this patient has already left the room^12^. We assume a maximum time $\tau_{max}$ after which a patient cannot be responsible for the colonization of another patient. To this end, we assume that a room’s infection status of strain $j$ decreases linearly in time from $Y_{\beta j}(t)=I_{j}(t_{j\beta}^{last})$ at $t=t_{j\beta}^{last}$ to $Y_{\beta j}(t)=0$ at $t=t_{j\beta}^{last}+\tau_{max}$.

Based on the considerations above, we define a time-dependent infection influence of patient $j$ on patient $i$ as

$$c_{i\leftarrow j}\left( t \right)=S_{i}\left( t \right)\sum_{\beta} F_{\alpha_{i}\left( t \right),\beta}Y_{\beta j}\left( t \right).$$

This time-dependent infection influence may lead to a total number of infectious contact time in days given by

$$C_{i\leftarrow j}=\int_{max(t_{i}^{adm},t_{i}^{test}-9d)}^{t_{i}^{test}-2d} S_{i}\left( t \right)\sum_{\beta} F_{\alpha_{i}\left( t \right),\beta}Y_{\beta j}\left( t \right)dt.$$

In this modeling approach, we can control the influence of the hospital structure using parameter $\xi$ and the influence of cleaning using parameter $\tau_{max}$.

The next step is to choose $\xi$ and $\tau_{max}$ which are best suitable for the hospital.

We set $\xi=1/10$, implying a strong impact of rooms and less impact of wards and higher hierarchy groups on the flux. This value reflects the experience that patients get colonized by VRE during their stay with colonized patients in a room. Furthermore, there is evidence that colonized pre-occupants lead to a colonization of subsequent patients.

The maximum amount of time, $\tau_{max}$, a patient may still be responsible for the infection of another is influenced by hygienic protocols of the hospital. Byers et al. claimed that an average of 2.8 disinfections is necessary to eradicate VRE from a hospital room. The observed hospital wards have a daily cleaning routine which is why we set $\tau_{max}$ to three days.

References:

1. 1. Vanhems P, Barrat A, Cattuto C, Pinton JF, Khanafer N, Regis C, et al. Estimating potential infection transmission routes in hospital wards using wearable proximity sensors. PloS one. 2013;8(9):e73970. Epub 2013/09/17. doi: 10.1371/journal.pone.0073970. PubMed PMID: 24040129; PubMed Central PMCID: PMCPMC3770639.
2. 2. Voirin N, Payet C, Barrat A, Cattuto C, Khanafer N, Regis C, et al. Combining high-resolution contact data with virological data to investigate influenza transmission in a tertiary care hospital. Infection control and hospital epidemiology. 2015;36(3):254-60. Epub 2015/02/20. doi: 10.1017/ice.2014.53. PubMed PMID: 25695165.
3. 3. Maier BF, Huepe C, Brockmann D. Modular hierarchical and power-law small-world networks bear structural optima for minimal first passage times and cover time. Journal of Complex Networks. 2019;7(6):865-95. doi: 10.1093/comnet/cnz010.
4. 4. Watts DJ, Dodds PS, Newman MEJ. Identity and Search in Social Networks. Science. 2002;296(5571):1302. doi: 10.1126/science.1070120.
